# Melatonin Synergizes With Mesenchymal Stromal Cells Attenuates Chronic Allograft Vasculopathy

**DOI:** 10.3389/fimmu.2021.672849

**Published:** 2021-04-29

**Authors:** Ya-fei Qin, De-jun Kong, Hong Qin, Yang-lin Zhu, Guang-ming Li, Cheng-lu Sun, Yi-ming Zhao, Hong-da Wang, Jing-peng Hao, Hao Wang

**Affiliations:** ^1^Department of General Surgery, Tianjin Medical University General Hospital, Tianjin, China; ^2^Tianjin General Surgery Institute, Tianjin Medical University General Hospital, Tianjin, China; ^3^Department of Anorectal Surgery, The Second Hospital of Tianjin Medical University, Tianjin, China

**Keywords:** chronic allograft vasculopathy, melatonin, mesenchymal stromal cells, aorta transplantation, mice

## Abstract

**Background:**

Chronic rejection characterized by chronic allograft vasculopathy (CAV) remains a major obstacle to long-term graft survival. Due to multiple complicated mechanisms involved, a novel therapy for CAV remains exploration. Although mesenchymal stromal cells (MSCs) have been ubiquitously applied to various refractory immune-related diseases, rare research makes a thorough inquiry in CAV. Meanwhile, melatonin (MT), a wide spectrum of immunomodulator, plays a non-negligible role in transplantation immunity. Here, we have investigated the synergistic effects of MT in combination with MSCs in attenuation of CAV.

**Methods:**

C57BL/6 (B6) mouse recipients receiving BALB/c mouse donor aorta transplantation have been treated with MT and/or adipose-derived MSCs. Graft pathological changes, intragraft immunocyte infiltration, splenic immune cell populations, circulating donor-specific antibodies levels, cytokine profiles were detected on post-operative day 40. The proliferation capacity of CD4^+^ and CD8^+^ T cells, populations of Th1, Th17, and Tregs were also assessed *in vitro*.

**Results:**

Grafts in untreated recipients developed a typical pathological feature of CAV characterized by intimal thickening 40 days after transplantation. Compared to untreated and monotherapy groups, MT in combination with MSCs effectively ameliorated pathological changes of aorta grafts indicated by markedly decreased levels of intimal hyperplasia and the infiltration of CD4^+^ cells, CD8^+^ cells, and macrophages, but elevated infiltration of Foxp3^+^ cells. MT either alone or in combination with MSCs effectively inhibited the proliferation of T cells, decreased populations of Th1 and Th17 cells, but increased the proportion of Tregs *in vitro*. MT synergized with MSCs displayed much fewer splenic populations of CD4^+^ and CD8^+^ T cells, Th1 cells, Th17 cells, CD4^+^ central memory T cells (Tcm), as well as effector memory T cells (Tem) in aorta transplant recipients. In addition, the percentage of splenic Tregs was substantially increased in the combination therapy group. Furthermore, MT combined with MSCs markedly reduced serum levels of circulating allospecific IgG and IgM, as well as decreased the levels of pro-inflammatory IFN-γ, TNF-α, IL-1β, IL-6, IL-17A, and MCP-1, but increased the level of IL-10 in the recipients.

**Conclusions:**

These data suggest that MT has synergy with MSCs to markedly attenuate CAV and provide a novel therapeutic strategy to improve the long-term allograft acceptance in transplant recipients.

## Introduction

Chronic rejection is supposed to be the principal obstacle to affect long-term graft or patients survival in most transplantation cases (1). The main pathological manifestation of chronic rejection is chronic allograft vasculopathy (CAV), characterized by neointimal proliferation, interstitial inflammation, parenchymal cell damage, interstitial fibrosis, and progressive narrowing of the arterial lumen, resulting in allograft dysfunction or even graft loss (2). Approximately 47.4% of allografts develop CAV during 10-year follow-up according to the International Society for Heart and Lung Transplantation (3). Although re-transplantation is an available option, currently there is no alternative novel therapy for the prevention of CAV. Therefore, CAV is considered an urgent and serious problem that needs to be solved in transplantation (4).

The immunological and non-immunological factors contributing to CAV have varied somewhat across this research area (5–7). Notably, a majority of intragraft T cells are memory T cell (Tm) phenotypes during the process of CAV (8). CD4^+^ Tm cells have been previously documented to lead to allograft rejection by providing assistance to activate donor-reactive CD8^+^ T cells for rapid development of direct cytotoxicity, and to B cells for alloantibody production (9–11). Furthermore, our previous research has shown that inhibiting CD4^+^ Tm infiltration by blocking OX40/OX40L pathway can obviously alleviate the severity of CAV and greatly prolonged allograft survival in a mouse cardiac transplantation model (8). Besides, the bulk of infiltrating cells in neointima and adventitia during the development of CAV were CD3^+^ cells, and the ratio of CD4^+^/CD8^+^ T cells was almost two (12). Activated CD4^+^ T cells can secrete IL-2 and IFN-γ cytokines to disrupt the structure of the extracellular matrix, deposit extracellular collagen, and promote the proliferation of fibroblasts, thereby ultimately leading to the development of CAV (13). Impressively, more CD4^+^ T helper (Th) cells and mononuclear cells are recruited into the neointima and secrete IL-1, IL-6, and TNF-α, and induce smooth muscle cell migration and proliferation in the internal elastic lamina, eventually developing CAV (14). It has been proposed that the Th1 phenotype/IFN-γ axis is one of the most important mediators of CAV through inducing macrophage activation and upregulating MHC II antigen expression that favors T cell allosensitization (15, 16). At present, although the current immunosuppressive treatment such as rapamycin and cyclosporin can dramatically prolong transplant survival, persistent immune and inflammatory responses to the MHC-mismatched transplants play a pivotal role in the development of CAV. Given the pathogenesis of CAV is multifactorial, the novel and effective therapeutic strategy should be explored.

Mesenchymal stromal cells (MSCs) are considered as “immune privileged cells” due to low expression of MHC-II as well as the absence of costimulatory molecules such as B7-1, B7-2, CD40, and CD40L on the cell surface (17, 18). They have been proposed as the promising “live” drugs being able to target the anti-donor immune response and prolong allograft survival in human and rodent studies, which may offer new insights for the prevention of CAV (19–21). Beneficial immunomodulatory effects of MSCs in downregulating the effector function of T cells, B cells, and their paracrine have been shown in experimental transplant models (22–24). Federica et al. have demonstrated that pretransplant portal vein infusion of MSCs induced tolerance which is associated with Treg expansion and compromised anti-donor Th1 activity in a mouse semiallogeneic heart transplant model (25). Similarly, we have recently reported that endometrial regenerative cells, a type of MSCs obtained from menstrual blood, are capable of alleviating CAV (26). Although the role of MSCs in immunomodulation is encouraging, concomitant immunosuppressants remain a challenge. Some immunosuppressive agents can restrict MSCs viability or function (27). For example, the beneficial role of MSCs will be antagonized by rapamycin and FK-506 *via* decreasing cell viability, differentiation, and proliferation (28–30). Consequently, additional consideration should be given when the regimens are chosen for the combination therapy with MSCs. More impressively, the life span of MSCs is severely restricted by harsh microenvironment *in vivo* leading to more than 80–90% of implanted cells being dead within 72 h after injection (31). The excessive reactive oxygen species (ROS) induced by ischemia-reperfusion injury and operation make further efforts to the apoptosis of MSCs (31). Given that all, a novel immunomodulator, which meanwhile act as an MSCs protector is urgently needed.

Melatonin (MT), also named N-acety-1-5-methoxytryptamine, is a neurohormone that is primarily known as the mediator for circadian rhythms, it also presents immunomodulatory, antioxidant, and anti-aging properties (32). It has been reported that treatment with high-dose MT (200 mg/kg/day) significantly prolonged rat cardiac allograft survival (33). Recipients receiving high-dose MT showed a marked decline in circulating allospecific IgG and IgM and lymphocyte proliferative capacity. Furthermore, high-dose MT is believed to prolong the survival of syngeneic islet grafts by inhibiting the proliferation of Th1 cells and enhancing the level of IL-10 (34). Enhancement of the graft function and immunological compliance were also observed in experimental transplantation of liver, lungs, and kidneys (35–37). In addition to its immunomodulatory effects, MT also serves as a cell protector, which protects MSCs from oxidation, inflammation, apoptosis when they are applied as a combination therapy (38). Besides, MT is consumed by human as a dietary complement without side-effects (39).

Given the extensive immunological and non-immunological properties of MT and MSCs, we hypothesized that MT synergizes with MSCs to attenuate CAV in the mouse aorta transplantation model.

## Materials and Methods

### Animals

Male adult BALB/C (H-2^d^) and C57BL/6 (B6, H-2^b^) mice (China Food and Drug Inspection Institute, Beijing, China) weighing 25–30 g were used as donors and recipients, respectively. All the mice were housed in a specific pathogen-free environment with the appropriate temperature, a 12 h dark/12 h light cycle, total nutrition feed, and clean water. All animal experimental procedures were approved by the Animal Care and Use Committee of Tianjin Medical University (Tianjin, China), and all the experiments were performed in accordance with the guideline of the Chinese Council on Animal Care.

### Adipose-Derived MSCs Harvest and Phenotype Identification

Adipose-derived MSCs (ADMSCs) were prepared according to the protocols described previously (40). Adipose tissue obtained from B6 mice was cut into < 1 mm^3^ pieces and then digested with 0.1% type I collagenase (1 mg/ml, Solarbio, Beijing, China) on the shaker (200 rpm) at 37°C for 60 min. The centrifuge was at 1,500 rpm for 5 min and the supernatant was discarded while retaining the sediment at the bottom of the centrifuge tube. After washing twice with PBS, the sediment was then resuspended in α-MEM complete medium (Hyclone, USA) with 15% fetal bovine serum (FBS, Hyclone, USA) and 1% penicillin/streptomycin (Solarbio, Beijing, China) and seeded in 6 cm plates. Then, the ADMSCs were cultured in a 37°C, 5% CO_2_ incubator. After 6 days of incubation, the cells displayed a fibroblast-like morphology. Subsequently, the third generation ADMSCs were collected for the identification of surface markers (CD29, CD34, CD45, SCA-1, CD44) (41) through flow cytometry.

### Orthotopic Aorta Transplantation and Experimental Groups

The recipient B6 and paired donor BALB/c mice were randomly divided into five experimental groups (n = 10 for each group): Group A, sham control group; Group B, untreated group; Group C, MT treated group; Group D, MSC treated group; Group E, MSC and MT combination treated group. Aorta transplantation, transplanting BALB/c mice aorta to B6 mice, was performed as described previously (26, 42). The recipients receiving a single dose of 1 × 10^6^ MSCs 1 day before transplantation and on postoperative days (PODs) 1, 7, 14, 21, 28, and 35 through tail vein injection. Considering impressive immunomodulatory properties of high dose MT (200 mg/kg/day) on acute allograft rejection, the same intervention was adopted (33). MT (HY-B0075, Med Chem Express, USA) accurately weighed according to body weight of mice was dissolved in 50 ul DMSO and then suspended in 450 ul saline solution and administered through a subcutaneous injection. MT was daily administered *via* subcutaneous injection in recipients from 1 day before transplantation to the end-stage of the study. Briefly, ketamine/xylazine cocktail (80 mg/kg ketamine, 4 mg/kg xylazine, i.p.) was used for anesthesia in combination with buprenorphine (0.05 mg/kg, s.c.) for analgesia. The mice were sacrificed with an overdose of the same anesthetic at the end point of the study. Grafts and spleens were harvested for evaluation at POD 40 (26). Meanwhile, the blood samples collected on POD 40 from the tail vein were centrifuged, and the sera were stored at −80°C freezer.

### Histological Assessment and Morphometric Analysis

The grafts (n = 10 for each group) were removed on POD 40 and fixed with 10% paraformaldehyde, then dehydrated for paraffin embedding, and cut into 4 μm slides. Hematoxylin and Eosin staining (H&E, G1120, Solarbio) were performed. The severity of CAV was determined by the degree of intimal hyperplasia and lumen occlusion. The scoring system, as previously described, was indicated as lumen occlusion (%) = intima/(intima+lumen). In brief, the score of 0, 1, 2, 3, 4, 5 point represents less than 10, 10–20, 20–40, 40–60, 60–80, 80–100% of lumen occlusion, respectively (43).

### Immunohistochemistry

To identify the intragraft infiltration of CD4^+^ and CD8^+^ cells, specific markers were stained for grafts (n = 10 for each group). Briefly, 0.01 mol/L sodium citrate buffer was used on tissue sections for antigen repair. The endogenous peroxidase was eliminated through incubation with 3% hydrogen peroxide for 25 min. Then the sections were incubated with rabbit anti-mouse CD4 antibody (diluted at 1:1,000, ab183685, Abcam, Cambridge, UK), rabbit anti-mouse F4/80 antibody (diluted at 1:1,000, ab100790, Abcam, Cambridge, UK), rabbit anti-mouse CD8 antibody (diluted at 1:2,000, ab217344, Abcam, Cambridge, UK), or rabbit anti-mouse Foxp3 antibody (diluted at 1:1,000, ab215206, Abcam, Cambridge, UK), overnight at 4°C and then incubated with 100 μl enhanced enzyme-labeled goat anti-rabbit IgG polymer (DAB kit, PV9000, ZSGB-BIO, Beijing, China) for 20 min next day at room temperature. Finally, a freshly prepared DAB solution was used to visualize the labeling. Non-specific staining was determined according to the negative control. The ImageJ (version 1.53, National Institutes of Health, USA) software was applied to quantify different cell types infiltration.

### Enzyme-Linked Immunosorbent Assay

The sera were obtained from the blood of all recipients after centrifuge (2,500 rpm for 8 min). Commercial ELISA kits were used to measure the levels of IL-10 (1211002, DAKEWE, Beijing, China), IL-17A (1211702, DAKEWE, Beijing, China), IL-6 (1210602, DAKEWE, Beijing, China), TNF-α (1217202, DAKEWE, Beijing, China), IL-1β (1210122, DAKEWE, Beijing, China), MCP-1 (1217392, DAKEWE, Beijing, China), and IFN-γ (1210002, DAKEWE, Beijing, China). All experimental operations were conducted according to the manufacturer’s protocols.

### Real-Time Polymerase Chain Reaction (RT-PCR)

The aorta allografts were harvested at POD 40 for detection of the key cytokines in the process of CAV. Total RNA was extracted using an RNAprep Pure Tissue Kit (DP 431, TIANGEN BIOTECH, Beijing, China, http://www.tiangen.com). The concentration and purity of the extracted RNAs were measured using a UV spectrophotometer. Total RNA was reverse transcribed into cDNA for expression analysis by using the FastKing gDNA Dispelling RT supermix kit (KR118, TIANGEN BIOTECH, Beijing, China). RT-PCR was performed using the 2×SYBR Green qPCR Master Mix (B21203, TIANGEN BIOTECH, Beijing, China) according to the manufacturer’s protocol. Primer sequences used in this experiment are listed in **Table 1**. The housekeeping GADPH was used as the normalization control. Relative differences of gene expression among the groups were calculated using the formula 2^−ΔΔCT^.

**Table 1:** The primer sequences used for real-time PCR.

Gene	Primers (5ʹ-3ʹ)
GADPH	Forward: AGGTCGGTGTGAACGGATTTG
	Reverse: TGTAGACCATGTAGTTGAGGTCA
IFN-γ	Forward: ATGAACGCTACACACTGCATC
	Reverse: CCATCCTTTTGCCAGTTCCTC
TNF-α	Forward: GATGGGGGGCTTCCAGAACT
	Reverse: GATGGGGGGCTTCCAGAACT
IL-1β	Forward: GAAGAGCCCATCCTCTGTGA
	Reverse: GGGTGTGCCGTCTTTCATTA
IL-6	Forward: TGACAACCACGGCCTTCCCTA
	Reverse: TCAGAATTGCCATTGCACAACTCTT
IL-17A	Forward: TTTAACTCCCTTGGCGCAAAA
	Reverse: CTTTCCCTCCGCATTGACAC
MCP-1	Forward: TTAAAAACCTGGATCGGAACCAA
	Reverse: GCATTAGCTTCAGATTTACGGGT
IL-10	Forward: ACTTCCCAGTCGGCCAGAGCCACAT
	Reverse: GATGACAGCGCCTCAGCCGCATCCT

### Splenocytes Co-culture With MT and/or MSCs *In Vitro*

In order to identify the role of MT and/or MSCs on CD4^+^ T cells, CD8^+^ T cells, Th1 cells, Th17 cells, and Tregs, a co-culture system was set *in vitro*, in which the experiments were performed using B6 splenocytes in the presence or absence of MT and/or MSCs in a 24-well plate. To activate and culture T cells, CD3 and CD28 functional antibodies (CD3, 1 ug/ml, CD28 2 ug/ml, eBioscience, San Diego, CA, USA) and IL-2 (2 ng/ml, PeproTech, NJ, USA) were used in the co-culture system. In the negative control group, neither MT nor MSCs was added into activated splenocytes. Meanwhile, activated splenocytes were co-cultured with MT and/or MSCs respectively. Briefly, splenocytes (1 × 10^6^ cells per well) were pretreated with MT (100 ug/ml) and/or MSCs (1 × 10^5^ cells per well) 2 h before adding the stimulators according to the previous study (44, 45). The splenocytes were stimulated with corresponding stimulators for 96 h with RPMI 1640 (HyClone, GE, USA) medium containing 10% FBS (HyClone, Thermo, USA) in a 37°C and 5% CO_2_ culture environment. After 96 h, the MSCs adhered to the surface of the culture dishes. Then the dishes were washed with RPMI medium and the suspended splenocytes were removed by aspiration with a pipette (46). The proliferation capacity of CD4^+^ and CD8^+^ T cells was evaluated by Ki67 staining through flow cytometry. In addition, Th1, Th17, and Tregs were also analyzed by flow cytometry.

### Determination of Circulating Donor-Specific Antibodies (DSA)

To determine the levels of DSA, 5 μl serum was collected from all the recipients on POD 40. Serum was diluted 20 times with PBS and co-cultivated with the splenocytes (5 × 10^5^ cells per well) obtained from the BALB/c at 37°C for 30 min, followed by staining with both anti-IgG-PE and anti-IgM-PE antibodies. The levels of DSA were measured by flow cytometry and presented as mean fluorescence intensity (MFI) (4).

### Flow Cytometry Analysis

The spleens were obtained from different groups after sacrifice. And the splenocytes were stained with different specific markers according to the previous study (8, 47). Th1 (CD4^+^IFN-γ^+^), Treg (CD4^+^CD25^+^Foxp3^+^), Th17 (CD4^+^IL-17A^+^), CD4^+^ central memory T cell (Tcm, CD4^+^CD44^high^CD62L^high^), and effector memory T cell (Tem, CD4^+^CD44^high^CD62L^low^) were stained with corresponding antibodies, including anti-CD4-FITC, anti-Foxp3-APC, anti-IFN-γ-PE, anti-IL-4-APC, anti-IL-17A-perCP, anti-CD62L-perCP, anti-CD44-APC. The detailed procedure of staining was the same as that previously described (48). All fluorescent-labeled antibodies, FcR blockers, and intracellular staining reagents for flow cytometry were purchased from either eBioscience (eBioscience, San Diego, CA, USA) or BioLegend (BioLegend, San Diego, CA, USA). The FlowJo (version 10.7.1, https://www.flowjo.com) software was applied to analyze the data.

### Statistical Analysis

All data were expressed as mean ± standard error of mean (SEM). Sample comparison between multiple groups was analyzed by one-way analysis of variance (ANOVA) after the normality test and followed by *post hoc* analysis with the least significant difference (LSD) test; P < 0.05 was considered statistically significant. SPSS (IBM SPSS Statistics version 22.0) software was used for statistical analysis.

## Results

### Characterization of MSCs

**Figure 1:**
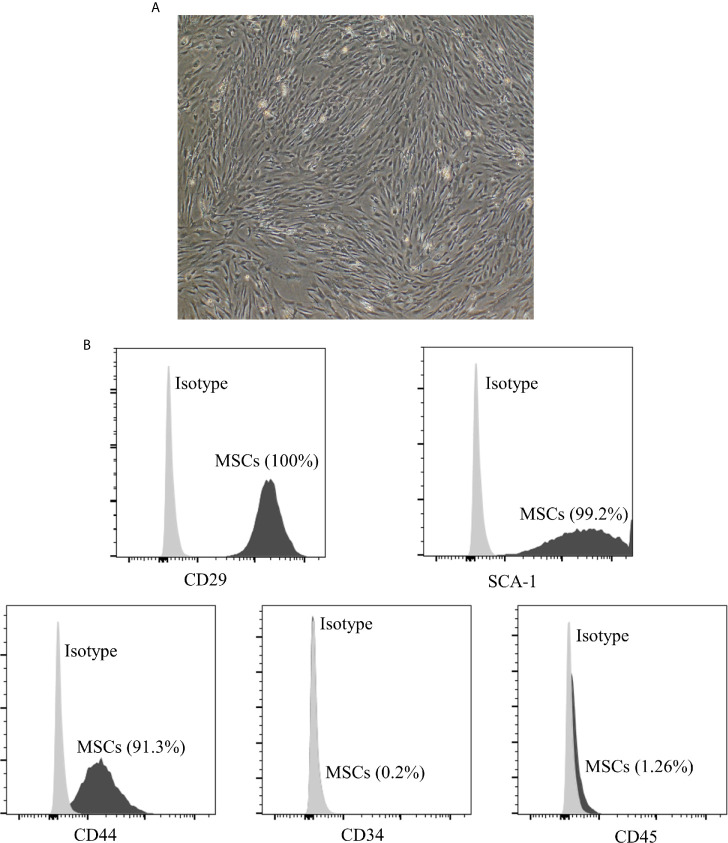
Characterization of adipose derived MSCs. **(A)** Morphology of p3 passage MSCs. The section is displayed at 100× magnification. The MSCs display a fibroblast-like or spindle-shaped morphology. **(B)** The expression of cell markers on the surface of MSCs measured by flow cytometry analysis. MSCs are positive for CD29 (100%), SCA-1 (99.2%), CD44 (91.3%), while negative for CD34 (0.2%) and CD45 (1.26%). MSCs, mesenchymal stromal cells.

To identify the phenotype of MSCs, the iconic markers of MSCs were determined by flow cytometry. As shown in **Figure 1**, MSCs displayed a fibroblast-like morphology and were positive for CD29 (100%), SCA-1 (99.2%), CD44 (91.3%), while negative for CD34 (0.2%) and CD45 (1.26%).

### MT Either Alone or Combined With MSCs Suppressed T Cell Proliferation *In Vitro*

**Figure 2:**
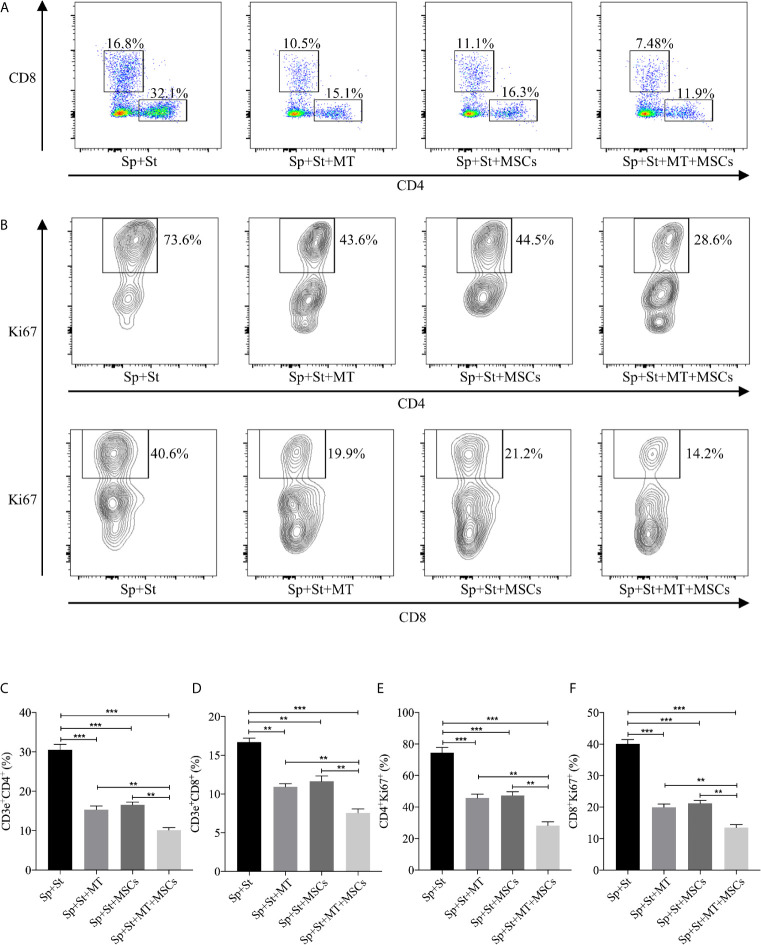
MT either alone or combined with MSCs suppresses T cell proliferation *in vitro*. The splenocytes derived from B6 mice are co-cultured with MT, MSCs, and MT+MSCs respectively in the presence of the stimulators of anti-CD3 Abs, anti-CD28 Abs and IL-2 *in vitro*. **(A)** The pseudocolor of CD4^+^ and CD8^+^ T cells *in vitro*; **(B)** Contour plot of CD4^+^Ki67^+^ and CD8^+^Ki67^+^ T cells *in vitro*; **(C)** The percentage of CD4^+^ T cells; **(D)** The percentage of CD8^+^ T cells; **(E)** The percentage of CD4^+^Ki67^+^ T cells; **(F)** The percentage of CD8^+^Ki67^+^ T cells. Statistical analysis is performed by one-way analysis of variance (ANOVA), ^**^ *p* <.01 and ^***^ *p* <.001. Bar graphs represent mean ± SEM. MT, melatonin; MSCs, mesenchymal stromal cells; Sp, splenocytes; St, stimulators. The assay was conducted three times with three replicates each time.

It has been previously demonstrated that MSCs could inhibit the proliferation of T cells. However, whether this effect can be augmented by MT remains unknown (49). The splenocytes obtained from B6 mice were stimulated with anti-CD3 Abs, anti-CD28 Abs, and IL-2, and co-cultured with MT, MSCs, and MT+MSCs, respectively *in vitro*. As shown in **Figures 2A, C, D**, the population of CD4^+^ and CD8^+^ T cells declined obviously in MT treated group (*vs.* Sp+St group: CD4, *p* <.001; CD8, *p* <.01). Moreover, the proportions further decreased strikingly after co-culture with MT+MSCs (*vs.* Sp+St group: CD4, *p* <.001; CD8, *p* <.001; *vs.* Sp+St+MT group: CD4, *p* <.01; CD8, *p* <.01; *vs.* Sp+St+MSCs group: CD4, *p* <.01; CD8, *p* <.01).

Ki67, a nuclear cell proliferation-associated antigen, was also stained to reflect T cell proliferation rate. When compared with untreated group, the proliferation of CD4^+^ and CD8^+^ T cells was significantly inhibited by MT treatment (**Figures 2B, E, F**, *vs.* Sp+St group: CD4, *p* <.001; CD8, *p* <.001). Additionally, MT+MSCs further inhibited CD4^+^ and CD8^+^ T cell proliferation (*vs.* Sp+St group: CD4, *p* <.001; CD8, *p* <.001; *vs.* Sp+St+MT group: CD4, *p* <.01; CD8, *p* <.01; *vs.* Sp+St+MSCs group; CD4, *p* <.01; CD8, *p* <.01). Given together, these results showed that MT either alone or in combination with MSCs could obviously decrease the proportion of CD4^+^ and CD8^+^ T cells and significantly inhibit T cell proliferation *in vitro.*

### MT Either Alone or Combined With MSCs Reduced Th1 and Th17 Population While Promoting Tregs *In Vitro*

**Figure 3:**
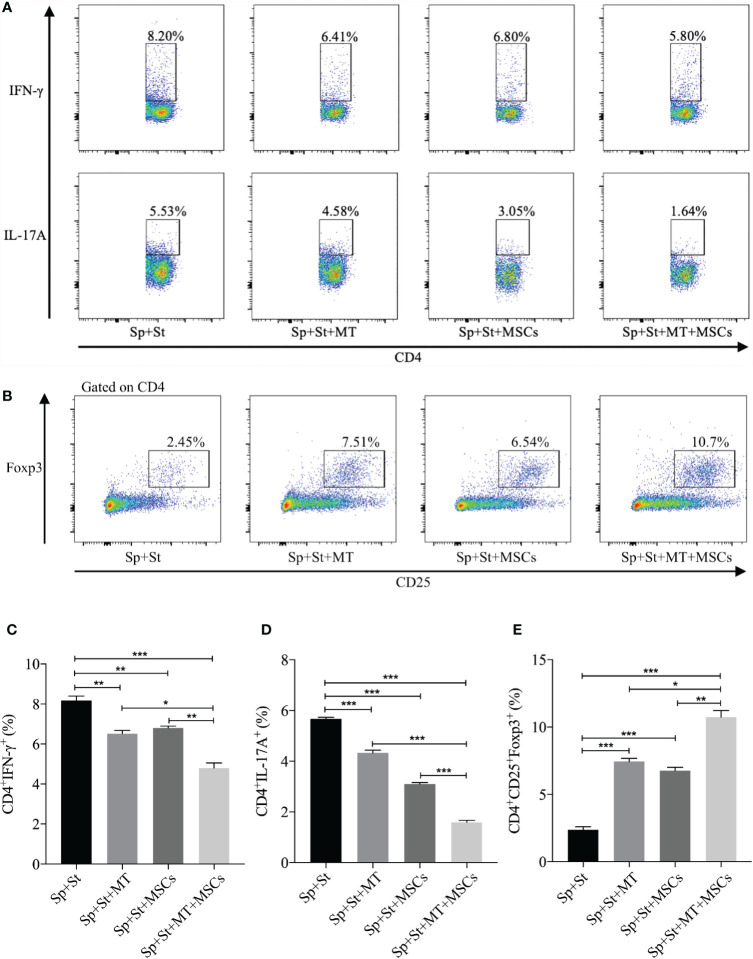
MT either alone or combined with MSCs reduces Th1 and Th17 populations while promoting Treg population *in vitro*. The splenocytes derived from B6 mice were co-cultured with MT, MSCs, and MT+MSCs respectively in the presence of the stimulators of anti-CD3 Abs, anti-CD28 Abs, and IL-2 *in vitro*. **(A)** The pseudocolor of Th1 (CD4^+^IFN-γ^+^) cells and Th17 (CD4^+^IL-17A^+^) cells *in vitro*; **(B)** The pseudocolor of Tregs (CD4^+^CD25^+^Foxp3^+^) *in vitro*; **(C)** The percentage of Th1 cells; **(D)** The percentage of Th17 cells; **(E)** The percentage of Tregs. Statistical analysis is performed by one-way analysis of variance (ANOVA), ^*^ *p* <.05, ^**^ *p* <.01, and ^***^ *p* <.001. Bar graphs represent mean ± SEM. MT, melatonin; MSCs, mesenchymal stromal cells; Sp, splenocytes; St, stimulators. The assay was conducted three times with three replicates each time.

CD4^+^ Th cells are considered to be the vital driving factors in the process of CAV (50, 51). In this experiment, the percentage of Th1 and Th17 cells were detected *in vitro*. Compared to negative control group, both CD4^+^IFNγ^+^ and CD4^+^IL-17A^+^ cells were decreased after co-culture with MT (**Figures 3A, C, D**, *vs.* Sp+St group: Th1, *p* <.01; Th17, *p* <.001), and further reduced in the combination group (*vs.* Sp+St group: Th1, *p* <.001; Th17, *p* <.001; *vs.* Sp+St+MT group: Th1, *p* <.05; Th17, *p* <.001; *vs.* Sp+St+MSCs group: Th1, *p* <.01; Th17, *p* <.001). On the contrary, Tregs could hamper the immune reaction of Th1 and Th17 cells. Therefore, the effect of MT and MSCs on regulating CD4^+^CD25^+^Foxp3^+^ T cells were also evaluated. As shown in **Figures 3B, E**, the percentage of CD4^+^CD25^+^Foxp3^+^ T cells was increased in MT (*vs.* Sp+St group, *p* <.001) or MSC monotherapy group. And the proportion tended to be the highest in combination treatment group (*vs.* Sp+St group, *p* <.001; *vs.* Sp+St+MT group, *p* <.05; *vs.* Sp+St+MSCs group, *p* <.01). This result suggests that MT synergizes with MSCs can significantly reduce Th1 and Th17 population, but augment Treg population *in vitro.*

### MT Synergized With MSCs Significantly Ameliorate CAV

**Figure 4:**
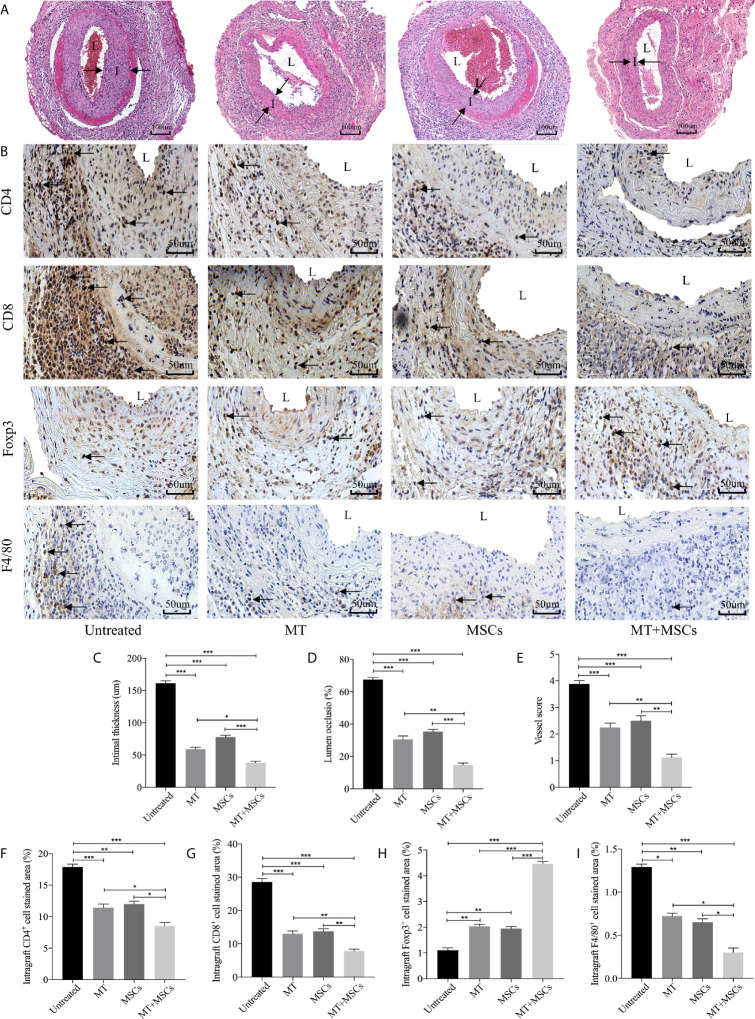
MT synergized with MSCs significantly ameliorates CAV. Each section is displayed at 400× magnification. “I” indicates neointima. “L” indicates lumen. **(A)** Histology of aorta allografts in transplant recipients; **(B)** Graft sections for immunohistochemical staining of CD4^+^ cells, CD8^+^ cells, Tregs and macrophages; **(C)** Intimal thickness of the aorta allografts in different groups; **(D)** Lumen occlusion of the aorta allografts in different groups; **(E)** Vessel score of the aorta allografts in different groups; **(F)** The percentage of intragraft CD4^+^ cells; **(G)** The percentage of intragraft CD8^+^ cells; **(H)** The percentage of intragraft Tregs; **(I)** The percentage of intragraft macrophages. Statistical analysis is performed by one-way analysis of variance (ANOVA), n = 10 per group, ^*^ *p* <.05, ^**^ *p* <.01, and ^***^ *p* <.001. Bar graphs represent mean ± SEM. MT, melatonin; MSCs, mesenchymal stromal cells. The experiments were repeated three times independently.

Based on the striking inhibition effect of MT and/or MSCs on CD4^+^ T and CD8^+^ T cells activation *in vivo*, we then assessed the role of MT and MSCs in the allogeneic aorta transplantation model. We have previously reported that aorta allografts collected at POD 40 developed typical features of vasculopathy in mice (52). In this study, **Figure 4A** showed that there was obvious lumen occlusion accompanied by the thickest neointima in aorta allografts of untreated mice. Either MT or MSC monotherapy could ameliorate intimal thickening of aorta grafts. Furthermore, when MT was added to MSC treatment, the vasculopathy was effectively inhibited in aorta allografts. As shown in **Figure 4C**, the intimal hyperplasia of aorta allografts was decreased by the treatment of either MT (*vs.* untreated group, *p* <.001) or MSCs, and further attenuated by the combination therapy (*vs.* untreated group, *p* <.001; *vs.* MT alone group, *p* <.05; *vs.* MSC alone group, *p* <.001). Additionally, we measured the intima/(intima+lumen) ratio in the grafts among different groups. As shown in **Figure 4D**, minimal intima/(intima+lumen) ratio was found in the MT+MSC group (*vs.* untreated group, *p* <.001; *vs.* MT alone group, *p* <.01; *vs.* MSC alone group, *p* <.001). Moreover, the vessel score, indicating the severity of CAV, was also the lowest in the combination therapy group (**Figure 4E**, *vs.* untreated group, *p* <.001; *vs.* MT alone group, *p* <.01; *vs.* MSC alone group, *p* <.01). Taken together, these data indicate that MT has a synergistic effect with MSCs to prevent the progression of CAV.

### MT Either Alone or Combined With MSCs Decreased CD4^+^ Cell, CD8^+^ Cell, and Macrophage Infiltration but Augmented Treg Infiltration in Aorta Allografts

To determine the intragraft infiltration of immune cells, immunohistology staining of CD4^+^ and CD8^+^ cells was performed. As shown in **Figures 4B, F–I**, a large number of CD4^+^ and CD8^+^ cells were localized in the graft neointima of untreated mice. Both CD4^+^ and CD8^+^ cells were decreased in either MT (*vs.* untreated group, CD4, *p* <.001; CD8, *p* <.001) or MSC treated group, and further dramatically reduced in the combination therapy group (*vs.* untreated group, CD4, *p* <.001; CD8, *p* <.001; *vs.* MT alone group: CD4, *p* <.05; CD8, *p* <.01; *vs.* MSC alone group: CD4, *p* <.05; CD8, *p* <.01). Conversely, MT effectively increased intragraft Treg infiltration (*vs.* untreated group, *p* <.01). Furthermore, MT synergizes with MSCs achieved highest infiltration level of Tregs in aorta allografts (*vs.* untreated group, *p* <.001; *vs.* MT group, *p* <.001; *vs.* MSC group, *p* <.001). Given that macrophages play an important role in the development of CAV, we sought to observe the infiltration of macrophages in the grafts. Either MT (*vs.* untreated group, *p* <.05) or MSC monotherapy could reduce intragraft macrophage infiltration. Notably, the lowest level of macrophages was detected in the combination therapy group (*vs.* untreated group, *p* <.001; *vs.* MT group, *p* <.05; *vs.* MSC group, *p* <.05). The above results indicate that synergistic effects of MT and MSCs in attenuating CAV are associated with the reduced infiltration of CD4^+^ cells, CD8^+^ cells, and macrophages, but increased infiltration of Tregs in the allografts.

### MT Acted Synergistically With MSCs to Reduce Splenic CD4^+^ and CD8^+^ T Cells, Inhibit B Cell Activation and DSA Production

**Figure 5:**
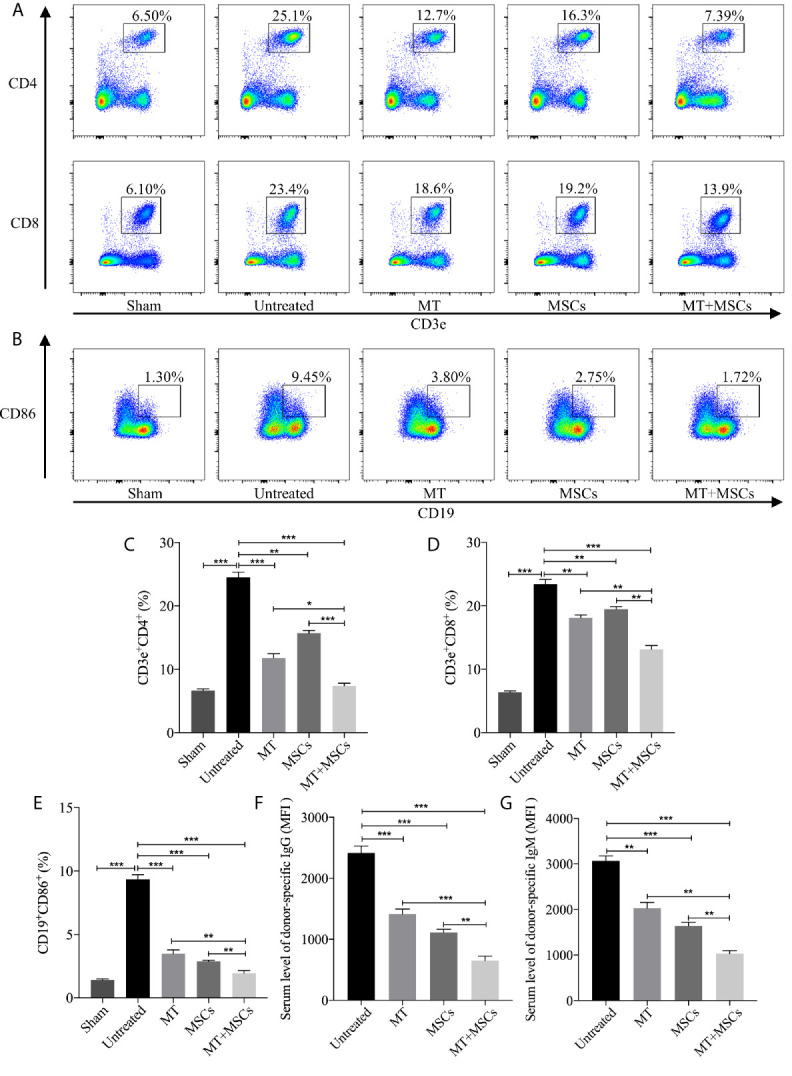
MT acts synergistically with MSCs to reduce splenic CD4^+^ and CD8^+^ T cells, inhibit B cell activation and DSA production. Splenocytes are collected from the B6 recipient mice of each group at postoperative day 40. **(A)** The pseudocolor of CD4^+^ and CD8^+^ T cells in recipient splenocytes; **(B)** The pseudocolor of B (CD19^+^CD86^+^) cells *in vivo*; **(C)** The percentage of CD4^+^ T cells; **(D)** The percentage of CD8^+^ T cells; **(E)** The percentage of B cells; **(F)** The serum level of donor-specific IgG; **(G)** The serum level of donor-specific IgM. Statistical analysis is performed by one-way analysis of variance (ANOVA), n = 10 per group, ^*^ *p* <.05, ^**^ *p* <.01, and ^***^ *p* <.001. Bar graphs represent mean ± SEM. MT, melatonin; MSCs, mesenchymal stem cells; MFI, mean fluorescence intensity. Data shown are representative of three separate experiments.

We have found the obviously decreased proliferation propriety and proportion of CD4^+^ and CD8^+^ T cells *in vitro.* Therefore, the systemic levels of CD4^+^ and CD8^+^ T cells in recipients were also detected in this study. In **Figures 5A, C, D**, the percentage of CD4^+^ and CD8^+^ T cells were highly increased in untreated group (*vs.* sham group: CD4, *p* <.001; CD8, *p* <.001). However, the population of CD4^+^ and CD8^+^ T cells obviously declined in MT treated group (*vs.* untreated group: CD4, *p* <.001; CD8, *p* <.01). Moreover, the proportion further decreased strikingly in MT+MSC treated group (*vs.* untreated group: CD4, *p* <.001; CD8, *p* <.001; *vs.* MT alone group: CD4, *p* <.05; CD8, *p* <.01; *vs.* MSC alone group: CD4, *p* <.001; CD8, *p* <.01).

Meanwhile, the features of B cells in driving immune response including antigen presentation to T lymphocytes, transition into plasma cells, and generation of DSA cannot be ignored in the process of CAV. In this study, CD19^+^CD86^+^ B cells were measured among different groups and the results were shown in **Figures 5B, E**. Compared to the sham group, the proportion of CD19^+^CD86^+^ B cells increased significantly in untreated group (*vs.* sham group, *p* <.001). Phenomenally, the population of CD19^+^CD86^+^ B cells was decreased greatly in MT (*vs.* untreated group, *p* <.001) or MSC treated group and was further decreased in the combination group (*vs.* untreated group, *p* <.001; *vs.* MT alone group, *p* <.01; *vs.* MSC alone group, *p* <.01). In addition, DSA produced by plasma cells was also evaluated among groups. Splenocytes obtained from the BALB/c mice were co-cultured with diluted B6 recipient serum and then stained with anti-IgG-PE and anti-IgM-PE antibodies, respectively. As shown in **Figures 5F, G**, both donor-specific IgG and IgM were decreased in MT (*vs.* untreated group, IgG, *p* <.001; IgM, *p* <.01) or MSC (*vs.* untreated group, IgG, *p* <.001; IgM, *p* <.001) treated group, indicating that either MT or MSC treatment restrained the formation of DSA. And this inhibitory effect was further strengthened in the combination therapy group (*vs.* untreated group, IgG, *p* <.001; IgM, *p* <.001; *vs.* MT alone group: IgG, *p* <.001; IgM, *p* <.01; *vs.* MSCs alone group: IgG, *p* <.01; IgM, *p* <.01). These data indicated that MT acts synergistically with MSCs to attenuate CAV through inhibiting B cell activation and alleviating DSA production in the transplant recipients.

### MT Either Alone or Combined With MSCs Reduced Splenic Th1, Th17, CD4^+^ Tm Populations but Enhanced Treg Population in the Aorta Transplant Recipients

**Figure 6:**
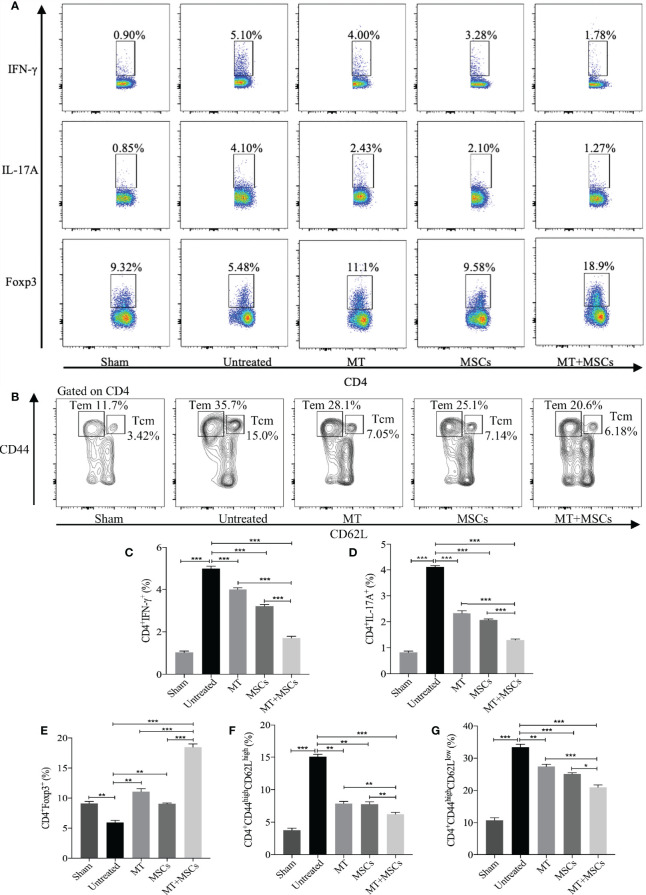
MT either alone or combined with MSCs reduces splenic Th1, Th17, CD4^+^ Tcm, and CD4^+^ Tem, but enhances Treg population in the aorta transplant recipients. Splenocytes are collected from the B6 recipient mice of each group at postoperative day 40. **(A)** The pseudocolor of Th1 (CD4^+^IFN-γ^+^) cells, Th17 (CD4^+^IL-17A^+^) cells, and CD4^+^Foxp3^+^ Tregs *in vivo*; **(B)** The contour plot of Tem (CD4^+^CD44^high^CD62L^low^) and Tcm (CD4^+^CD44^high^CD62L^high^) *in vivo*; **(C)** The percentage of Th1 cells; **(D)** The percentage of Th17 cells; **(E)** The percentage of Tregs; **(F)** The percentage of Tcm *in vivo*; **(G)** The percentage of Tem *in vivo*. Statistical analysis is performed by one-way analysis of variance (ANOVA), n = 10 per group, ^*^ *p* <.05, ^**^ *p* <.01, and ^***^ *p* <.001. Bar graphs represent mean ± SEM. MT, melatonin; MSCs, mesenchymal stromal cells. The experiments were independently repeated three times.

Based on the direct inhibitory effect of MT combined with MSCs on activation of Th1 and Th17 cells *in vitro*, we further determined whether MT and/or MSCs could modulate Th populations in the splenocytes of the aorta transplant recipients. Splenocytes of different groups were harvested and stained for Th1 (CD4^+^IFN-γ^+^) and Th17 (CD4^+^IL-17A^+^) cells. The percentage of Th1 and Th17 cells increased obviously in untreated group (*vs.* sham group: Th1, *p* <.001; Th17, *p* <.001). As compared with those of the untreated group, the percentages of both Th1 and Th17 cells were significantly decreased in MT (**Figure 6A**, Th1, *p* <.001; Th17, *p* <.001) or MSC group, and they were further reduced in the combination therapy group (*vs.* untreated group: Th1, *p* <.001; Th17, *p*<.001; *vs.* MT alone group: Th1, *p* <.001; Th17, *p* <.001; *vs.* MSC alone group: Th1, *p* <.001; Th17, *p* <.001).

In correlation with *in vitro* experiments and immunohistology staining, we have further measured the splenic Treg population in the recipients. As shown in **Figures 6A, C–E**, when compared with the untreated group, the percentage of splenic Tregs was obviously increased in MT (*vs.* untreated group, *p* <.01) or MSC treated recipients. And consistently, the Treg population was further increased significantly in the combined therapy group (*vs.* untreated group, *p* <.001; *vs.* MT alone group, *p* <.001; *vs.* MSC alone group, *p* <.001). This result suggested that MT combined with MSCs can modulate Th1, Th17, and Treg populations which are associated with attenuation of CAV in transplant recipients.

It has been proven that the blocking of CD4^+^ Tm cells (Tcm and Tem) could alleviate the severity of CAV (53). To identify whether MT and MSCs have synergistic effect in modulating CD4^+^ Tm, splenic Tcm (CD4^+^CD44^high^CD62L^high^) and Tem (CD4^+^CD44^high^CD62L^low^) were detected among different groups. The results were analyzed by flow cytometry, and the contour plot among the groups is shown in **Figures 6B, F, G**. As compared to sham group, the proportion of Tcm and Tem markedly increased in untreated group (*vs.* sham group, Tcm, *p* <.001; Tem, *p* <.001). However, the percentages of both Tcm and Tem were decreased in MT (*vs.* untreated group, Tcm, *p* <.01; Tem, *p* <.01) or MSC monotherapy group, and these two populations were further significantly reduced in combination therapy group (*vs.* untreated group, Tcm, *p* <.001; Tem, *p* <.001; *vs.* MT alone group: Tcm, *p* <.01; Tem, *p* <.001; *vs.* MSCs alone group: Tcm, *p* <.01; Tem, *p* <.05). These results suggested that Tm, which is critical to the pathogenesis of CAV, can be attenuated by the combination therapy of MT and MSC in allograft recipients.

### MT in Combination With MSCs Further Achieved the Reduced Levels of Pro-inflammatory Cytokines but Increased Level of IL-10 in Transplant Recipients

**Figure 7:**
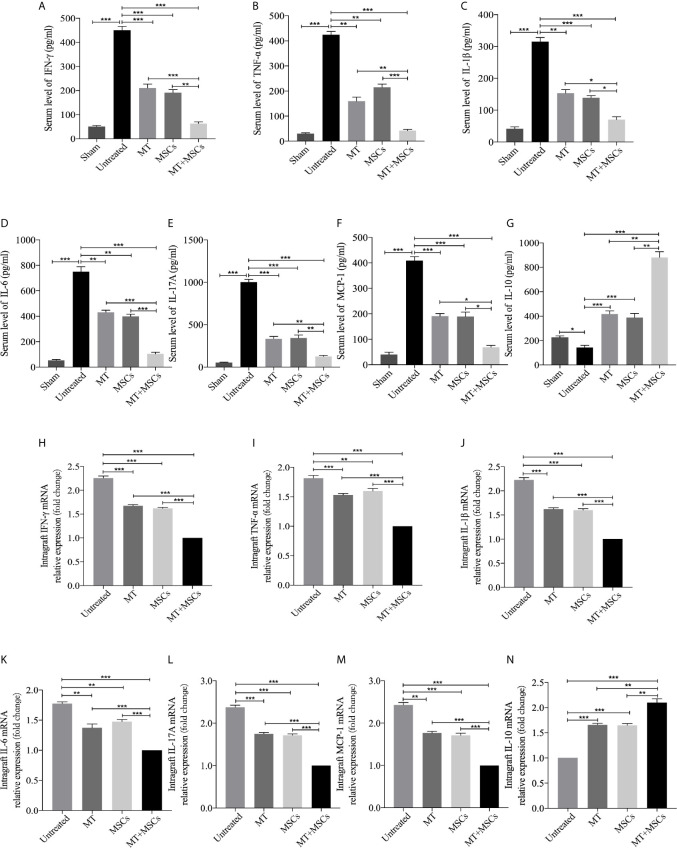
MT in combination with MSCs further achieves reduce levels of pro-inflammatory cytokines, but increased level of IL-10 in transplant recipients. The IFN-γ, TNF-α, IL-1β, IL-6, IL-17A, MCP-1, and IL-10 are detected the in the recipient sera and grafts. **(A)** The level of serum IFN-γ among the groups; **(B)** The level of serum TNF-α among the groups; **(C)** The level of serum IL-1β among the groups; **(D)** The level of serum IL-6 among the groups; **(E)** The level of serum IL-17A among the groups; **(F)** The level of serum MCP-1 among the groups; **(G)** The level of serum IL-10 among the groups; **(H)** The mRNA expression levels of IFN-γ among the groups; **(I)** The mRNA expression level of TNF-α among the groups; **(J)** The mRNA expression level of IL-1β among the groups; **(K)** The mRNA expression level of IL-6 among the groups; **(L)** The mRNA expression level of IL-17A among the groups; **(M)** The mRNA expression level of MCP-1 among the groups; **(N)** The mRNA expression level of IL-10 among the groups. Statistical analysis is performed by one-way analysis of variance (ANOVA), n = 10 per group, ^*^ *p* <.05, ^**^ *p* <.01, and ^***^ *p* <.001. Bar graphs represent mean ± SEM. MT, melatonin; MSCs, mesenchymal stromal cells. The experiments were independently repeated three times.

To further determine the cytokine profiles locally and systemically in the development of CAV, the levels of anti-inflammatory and pro-inflammatory cytokines were detected in both the grafts and the sera. As shown in **Figures 7A–G**, the serum levels of inflammatory cytokines were significantly increased in the untreated group (*vs.* sham group, IFN-γ, *p* <.001; TNF-α, *p* <.001; IL-1β, *p* <.001; IL-6, *p* <.001; IL-17A, *p* <.001; MCP-1, *p* <.001). As compared with those of untreated recipients, the serum levels of inflammatory cytokines (IFN-γ, TNF-α, IL-1β, IL-6, IL-17A, and MCP-1) were significantly decreased in the recipients receiving MT (*vs.* untreated group, IFN-γ, *p* <.001; TNF-α, *p* <.01; IL-1β, *p* <.01; IL-6, *p* <.01; IL-17A, *p* <.001; MCP-1, *p* <.001) or MSC monotherapy. Moreover, these cytokine levels were further reduced in the combination therapy group. In contrast, the serum level of IL-10 was significantly increased in MT (*vs.* untreated group, *p* <.001) or MSC monotherapy group, and it was further increased in the combination therapy group (*vs.* untreated group, *p* <.001; *vs.* MT alone group, *p* <.01; *vs.* MSC alone group, *p* <.01). Meanwhile, the levels of anti-inflammatory and pro-inflammatory cytokines were also examined in aorta allografts by RT-PCR. As shown in **Figures 7H-N**, similar to the results of serological tests, the intragraft levels of inflammatory cytokines were significantly increased in the untreated group. As compared with those of untreated recipients, the intragraft levels of inflammatory cytokines (IFN-γ, TNF-α, IL-1β, IL-6, IL-17A, and MCP-1) were significantly decreased in the recipients receiving MT (*vs.* untreated group, IFN-γ, *p* <.001; TNF-α, *p* <.001; IL-1β, *p* <.001; IL-6, *p* <.01; IL-17A, *p* <.001; MCP-1, *p* <.01) or MSC monotherapy. Moreover, these intragraft cytokine levels were further reduced in the combination therapy group. In contrast, the intragraft level of IL-10 was significantly increased in MT (*vs.* untreated group, *p* <.001) or MSC monotherapy group, and it was further increased in the combination therapy group (*vs.* untreated group, *p* <.001; *vs.* MT alone group, *p* <.01; *vs.* MSC alone group, *p* <.01). These data demonstrate that MT and MSCs act synergistically to ameliorate CAV by regulating cytokine profiles locally and systemically in the transplant recipients.

## Discussion

Chronic vasculopathy is the major cause of end-stage graft loss. It has been reported that infection, ischemic injury, oxidative stress, adaptive immune response, and other risk factors are linked to the development of CAV (51). As of yet, a novel therapy that directly targets chronic vasculopathy is still lacking. MSCs have been proposed as promising drugs due to their distinct immunomodulatory features. ADMSCs represent biological advantages in immunomodulatory effects, such as downregulating the effector function of dendritic cells and promoting the transfer of tolerogenic dendritic cells (54). Meanwhile, the immunomodulatory, antioxidant, and anti-apoptotic properties of MT have attracted extensive attention for its therapeutic application (32). Our study has, for the first time, provided strong evidence that MT has synergy with MSCs to effectively ameliorate graft pathological changes of CAV. The dosage of MT used in this study was based on the previous reports (33, 34). It has been previously reported that high-dose of MT (200 mg/kg/day) used for treatment of Parkinsonism had no any signs of serious adverse effects apart from transient sedation (55). In addition, no adverse effects were observed when the repeated high-dose of MT was applied intravenously to the recipients (56, 57). Furthermore, in the current study we have also demonstrated that the same dosage (200 mg/kg/day) of MT has no side-effects observed and is safe in transplant recipients.

Based on the evaluation of the infiltrating cells in the neointima and the endothelial layer of aorta allografts, it was observed that the majority of infiltrating cells were CD4^+^ and CD8^+^ cells (12) which were recognized as key contributors to CAV (58). *In vitro*, the population of CD4^+^ and CD8^+^ T cells declined obviously in the MT treated group and further decreased strikingly after co-culture with MT+MSCs. Meanwhile, CD4^+^ and CD8^+^ T cells proliferation rate was further detected according to Ki67 staining. We have also identified that the proliferation of CD4^+^ and CD8^+^ T cells were significantly inhibited by MT and/or MSCs *in vitro*. Consistently, it was found that the local (aorta allografts) and systemic (recipient spleens) levels of CD4^+^ and CD8^+^ T cells in the recipients were also significantly declined by the treatment of MT and/or MSCs. According to the literature reports, the expressions of T cell activating factors (CD3e, lck, ZAP 70, LAT, and slp76) were significantly decreased when the MT dosage was increased. Similarly, the expression of CD69, a classic marker of early lymphocyte and T cell activation was significantly higher than that of normal group when the level of internal MT dropped after 6 weeks (59). Meanwhile, the interplay between MSCs and T cells have been extensively investigated. The proliferation-inhibiting effect of MSCs on T cells is thought to be meditated by the release of transforming growth factor-beta (TGF-β) and hepatocyte growth factor (HGF), which leads to the decrease of cyclin D2 and increase of p27^kipl^ expression in T cells, resulting in arrest of proliferation in the G1 phase (60). The data obtained from our study have demonstrated that the inhibitory effect of MT and/or MSCs on the proliferation of CD4^+^ and CD8^+^ T cells is crucial for the prevention of CAV.

Th1 and its hallmark secretion factor IFN-γ play an essential role in initiating inflammatory response, thereby contributing to the development of CAV (61). IFN-γ has been demonstrated to play a vital role in destroying the structure of the extracellular matrix, facilitating the proliferation of vascular smooth muscle cells and the deposition of extracellular collagen, ultimately leading to the development of CAV (13). The grafts in IFN-γ^−/−^ mice have no signs of CAV for up to 8 weeks post-transplantation. In contrast, the grafts in wildtype recipients developed severe CAV in 50% of the recipients (62). There is increasing evidence that MSCs are capable of suppressing the expression of T-bet, a Th1-specific transcription factor and decreasing the level of IFN-γ (63). Furthermore, high-dose MT is believed to prolong the survival of syngeneic islet grafts by inhibiting the proliferation of Th1 cells (34). The above findings were further verified by our current study. We observed that infusion of both MT and MSCs to the recipients could synergistically inhibit Th1 cell response, thus preventing the progression of the CAV. Similarly, our *in vitro* co-culture experiments demonstrated that either MT or MSCs were capable of suppressing the activation of Th1 cells, and this inhibitory effect was further strengthened in the combination therapy group. In addition, MT in synergy with MSCs dramatically downregulated the level of IFN-γ in both the circulation and the allografts, and played a key role in the process of CAV.

IL-17, secreted by Th17 cells, is a potent proinflammatory cytokine that induces chemokine expression and leukocyte infiltration (64). The recent study suggested that, in addition to Th1 response, Th17 cell response exists in the process of CAV, and neutralization of IL-17 can ameliorate CAV (62). It comes to light that the infiltration of IL-17-producing CD4^+^ T cells are predominant in an established MHC II mismatched (bm12-to-B6) model accompanied by early vascular inflammation and CAV (62). Nuria et al. (65) have discovered that the peripheral and central Th17 responses were distinctly inhibited by MT in the immunized experimental autoimmune encephalomyelitis model. In the current study, we have also observed that MT could significantly decrease the population of Th17 and the serum level of IL-17 in aorta transplant recipients. The underlying interaction between RORα, a lineage-specific transcription factor for Th17 cells, and MT could be the potential mechanism for the inhibition of Th17 cell differentiation. RORα highly promotes the generation of Th17 from naive T cells, while a deficiency of RORα inhibits the expression of IL-17 *in vitro* and *in vivo* (66). The degradation of RORα or translocation of RORα from the nucleus induced by MT may lead to this inhibitory effect (67). Also, after binding to MT1 receptor, MT promotes the phosphorylation of Erk1/2, which in turn induces the activation of CAAT/enhancer-binding protein α (C/EBPα). C/EBPα binds to the promoter of REV-ERBα and inhibits the expression of REV-ERBα. Subsequently, REV-ERBα reduces the expression of NFIL3 by binding to a consensus sequence in the Nfil3 gene locus. The recruitment of NFIL3 to the promoters of rora and rorc gene inhibits the expression of RORα (67). A similar trend was observed in the MSC monotherapy group, which is consistent with our previous research concerning that Th17 response was inhibited by MSCs in acute cardiac allograft rejection (68). Novel findings have indicated that the inhibition of Th17 cells by MSCs is also mediated by HGF (69). Furthermore, it has been reported that MSCs impair the IL-17 production by Th17 cells in a contact-dependent manner (70). The decreased expressions of allograft IL-17A and splenic Th17 cells have suggested that MT has synergy with MSCs in inhibiting Th17 response.

Compared with naive T cells, CD4^+^ Tm cells have a lower activation threshold and are less dependent on co-stimulator molecules (71). Besides, the immune response of antigen-experienced CD4^+^ Tm is much faster and stronger than those of naive T cells during a second-time immune response (72). It has been shown that CD4^+^ Tm cells were a major population of infiltrating mononuclear cells in patients with CAV (12). Thus, alternative immunomodulatory protocols aiming to reduce CD4^+^ Tm response are required. In this study, we have demonstrated that the splenic population of both CD4^+^ Tcm and CD4^+^ Tem were reduced in recipients treated with MT and/or MSCs. This finding was correlated with the previous reports demonstrating that MSCs and MT could independently inhibit CD4^+^ Tcm and CD4^+^ Tem (65, 73, 74). To our knowledge, lacking expression of CD44 on the surface of splenic CD4^+^ T cells impairs the T cell adhesion to vascular endothelial cell (65, 75). Álvarez-Sánchez et al. have also reported that MT treatment could reduce CD44 expression in both peripheral Tem and Tcm, as well as downregulate the levels of IL-17A, IFN-γ, TNF-α in Tem (65). The results obtained from the present study demonstrated the reduced numbers of CD4^+^ Tcm and CD4^+^ Tem with a marked decrease in CD44 expression following the combination treatment of MT and MSCs, which are in line with the previous reports (65). On the basis of the *in vitro* data, we speculated that the sharp inhibition of CD4^+^ T cell proliferation is one of the causes for the decrease of CD4^+^ Tcm and CD4^+^ Tem. We believe that the low expressions of CD4^+^ Tcm and CD4^+^ Tem are associated with ameliorated pathological changes of CAV.

Tregs play a major role in maintaining immune balance and preventing CAV (48, 76). Here, we further evaluated the effect of the combination therapy on the regulation of Treg population. MT monotherapy distinctly increased the proportion of splenic Tregs in transplant recipients and promoted Foxp3^+^ Treg infiltration in allografts. Although MT caused a reduction in the number of Tregs in patients with metastatic solid tumors, it augments the number of CD3^+^CD4^+^Foxp3^+^ cells in SLE (77). Based on the present study, we believe that MT increased the formation of Tregs under an inflammatory status. The previous research by Zhao et al. assumed that the upregulation of the Tregs was due to the inhibition of calcium/calmodulin-dependent kinase IV (CAMKIV) by MT (78). CAMKIV has been considered as a putative MT target. Silencing of CAMKIV increases Foxp3 production upon TGF-β stimulation from untreated lupus patients, indicating that CAMKIV acts as a negative Tregs regulator (79). However, detailed mechanisms still need to be elaborated. Instead, it is known that the generation of Treg induced by MSCs is predominantly dependent on TGF-β1 and IL-10 cytokines (80). Likewise, in the present study, the IL-10 levels in both the sera and the allografts were increased in either MT or MSC monotherapy group. Thus, the elevated level of IL-10 could be a cause for Treg generation. Furthermore, we have also found that MT in synergy with MSCs prevented the development of CAV, suggesting that this finding is associated with the augment of Tregs.

DSA can activate complement and combine with intimal matrix and type IV collagen of endothelial cells, thus resulting in an unnational disorder of vascular endothelium and structural damage (81, 82). In the present study, we found that MT synergized with MSCs markedly inhibited allospecific IgG and IgM on POD 40. Based on the stimulating effect of CD4^+^ Tm on the alloantibody production of B cells, we consider that the decreased proportion of CD4^+^ Tcm and CD4^+^ Tem in the present study may contribute to the low level of DSA. However, we did not find significant differences in total IgM and IgG among the experimental groups (data not shown), indicating that both MT and MSCs can effectively inhibit the generation of donor-specific immunoglobulins. Similar to the tendency of DSA, the population of CD19^+^CD86^+^ B cells were dramatically decreased in the recipients when treated with MT and MSCs, suggesting that MT synergized with MSCs could block DSA formation and restrain the response of CD19^+^CD86^+^ B cells. Several lines of evidences have demonstrated that MSCs are capable of suppressing the proliferation, differentiation, and activation of plasma cell generation, immunoglobulin-secreting ability of B cells through soluble factors secretion and cell-cell contact (83). Similarly, it has been previously reported that the expression of B cell activating factors CD19 were downregulated when pinealectomy mice were treated with exogenous MT (59), and this finding is in concurrence with our results.

In this study, untreated group developed a typical pathological feature of CAV accompanying with markedly increased infiltration of CD4^+^ cells, CD8^+^ cells, and macrophages, as well as the upregulated populations of splenic Th1, Th17, and CD4^+^ Tm, and the enhanced levels of circulating allospecific IgG and IgM. In addition, our pilot study showed that these results had no significant differences between untreated group and DMSO vehicle control group (data not shown). Ultimately, we have demonstrated that either MT or MSC monotherapy could ameliorate the pathological changes of CAV. In addition to its immunomodulatory effects, MT could work through alleviating ischemia reperfusion and blocking oxidative stress of allografts. Therefore, it suggests that MT exerts its modulatory effect through both immunological and non-immunological properties. Moreover, we have elaborated that MT combined with MSCs dramatically abrogated allograft CAV, and the superposition of the effects of the two agents is warranted. Although the in-depth mechanisms and specific signaling pathways of MT in regulating chronic immune response still need to be elucidated, the present study highlights that MT has synergy with MSCs in suppressing CAV, and provides a novel therapeutic strategy for the prevention of chronic allograft rejection.

## Conclusion

In the present study, we have investigated synergistic effects of MT and MSCs in attenuation of CAV in a mouse aorta transplantation model. Our results have demonstrated that MT synergizes with MSCs to significantly suppress allogeneic Th1, Th17, and CD4^+^ Tm cell responses, decrease DSA production as well as promote the Treg population. These encouraging data were further convinced by obviously ameliorated pathological manifestations of CAV. Our study highlights the efficacy of MT and MSC combination therapy in inhibiting CAV and provides a novel therapeutic strategy to prevent CAV and thereby achieving long-term allograft survival following transplantation.

## Data Availability Statement

The original contributions presented in the study are included in the article/supplementary material. Further inquiries can be directed to the corresponding author.

## Ethics Statement

The animal study was reviewed and approved by the Animal Care and Use Committee of Tianjin Medical University.

## Author Contributions

Y-FQ: conception and design, collection and assembly of data, data analysis and interpretation, and manuscript writing. D-JK and HQ: collection and assembly of data, data analysis and interpretation, and manuscript writing. Y-LZ, G-ML, C-LS, Y-MZ, H-DW, and J-PH: collection of data, data analysis and interpretation. HW: conception and design, financial support, administrative support, manuscript writing, and final approval of the manuscript. All authors contributed to the article and approved the submitted version.

## Funding

This work was supported by grants to HW from the National Natural Science Foundation of China (No. 82071802), Tianjin Application Basis and Cutting-Edge Technology Research Grant (No. 14JCZDJC35700), Li Jieshou Intestinal Barrier Research Special Fund (No. LJS_201412), Natural Science Foundation of Tianjin (No. 18JCZDJC35800), and Tianjin Medical University Talent Fund; by a grant to D-JK from Tianjin Research Innovation Project for Postgraduate Students (No. 2019YJSS184); by a grant to G-ML from Tianjin Research Innovation Project for Postgraduate Students (2020YJSS176); and by a grant to H-DW from Tianjin Research Innovation Project for Postgraduate Students (2020YJSS177).

## Conflict of Interest

The authors declare that the research was conducted in the absence of any commercial or financial relationships that could be construed as a potential conflict of interest.

## References

[1] Justiz Vaillant AA Mohseni M. Chronic Transplantation Rejection. Statpearls. Treasure Island, FL: StatPearls Publishing Copyright © 2020, StatPearls Publishing LLC (2020). 30571056

[2] Taylor DO Edwards LB Aurora P Christie JD Dobbels F Kirk R. Registry of the International Society for Heart and Lung Transplantation: Twenty-Fifth Official Adult Heart Transplant Report–2008. J Heart Lung Transplant (2008) 27(9):943–56. doi: 10.1016/j.healun.2008.06.017 18765186

[3] Lund LH Khush KK Cherikh WS Goldfarb S Kucheryavaya AY Levvey BJ. The Registry of the International Society for Heart and Lung Transplantation: Thirty-fourth Adult Heart Transplantation Report-2017; Focus Theme: Allograft Ischemic Time. J Heart Lung Transplant (2017) 36(10):1037–46. doi: 10.1016/j.healun.2017.07.019 28779893

[4] Luo Z Liao T Zhang Y Zheng H Sun Q Han F. Triptolide Attenuates Transplant Vasculopathy Through Multiple Pathways. Front Immunol (2020) 11:612. doi: 10.3389/fimmu.2020.00612 32373115 PMC7186401

[5] Wang CY Aronson I Takuma S Homma S Naka Y Alshafie T. cAMP Pulse During Preservation Inhibits the Late Development of Cardiac Isograft and Allograft Vasculopathy. Circ Res (2000) 86(9):982–8. doi: 10.1161/01.res.86.9.982 10807871

[6] Banner NR Thomas HL Curnow E Hussey JC Rogers CA Bonser RS. The Importance of Cold and Warm Cardiac Ischemia for Survival After Heart Transplantation. Transplantation (2008) 86(4):542–7. doi: 10.1097/TP.0b013e31818149b9 18724223

[7] Antunes A Prieto D Pinto C Branco C Correia P Batista M. Coronary Allograft Vasculopathy After Cardiac Transplantation: Prevalence, Prognostic and Risk Factors. Rev Portuguesa Cirurgia Cardio-toracica e Vasc Orgao Oficial da Sociedade Portuguesa Cirurgia Cardio-Toracica e Vasc (2017) 24(3-4):158. 29701389

[8] Wang H Zhang Z Tian W Liu T Han H Garcia B. Memory T Cells Mediate Cardiac Allograft Vasculopathy and are Inactivated by Anti-OX40L Monoclonal Antibody. Cardiovasc Drugs Ther (2014) 28(2):115–22. doi: 10.1007/s10557-013-6502-9 PMC4539019 24254032

[9] Chen Y Heeger PS Valujskikh A. In Vivo Helper Functions of Alloreactive Memory CD4+ T Cells Remain Intact Despite Donor-Specific Transfusion and anti-CD40 Ligand Therapy. J Immunol (Baltimore Md 1950) (2004) 172(9):5456–66. doi: 10.4049/jimmunol.172.9.5456 15100287

[10] Zhang Q Chen Y Fairchild RL Heeger PS Valujskikh A. Lymphoid Sequestration of Alloreactive Memory CD4 T Cells Promotes Cardiac Allograft Survival. J Immunol (Baltimore Md 1950) (2006) 176(2):770–7. doi: 10.4049/jimmunol.176.2.770 16393960

[11] Zhang QW Rabant M Schenk A Valujskikh A. Icos-Dependent and -Independent Functions of Memory CD4 T Cells in Allograft Rejection. Am J Transplant (2008) 8(3):497–506. doi: 10.1111/j.1600-6143.2007.02096.x 18294146

[12] van Loosdregt J van Oosterhout MF Bruggink AH van Wichen DF van Kuik J de Koning E. The Chemokine and Chemokine Receptor Profile of Infiltrating Cells in the Wall of Arteries With Cardiac Allograft Vasculopathy is Indicative of a Memory T-helper 1 Response. Circulation (2006) 114(15):1599–607. doi: 10.1161/circulationaha.105.597526 17015796

[13] Pichler M Rainer PP Schauer S Hoefler G. Cardiac Fibrosis in Human Transplanted Hearts is Mainly Driven by Cells of Intracardiac Origin. J Am Coll Cardiol (2012) 59(11):1008–16. doi: 10.1016/j.jacc.2011.11.036 22402073

[14] Chih S Chong AY Mielniczuk LM Bhatt DL Beanlands RS. Allograft Vasculopathy: The Achilles’ Heel of Heart Transplantation. J Am Coll Cardiol (2016) 68(1):80–91. doi: 10.1016/j.jacc.2016.04.033 27364054

[15] Nagano H Mitchell RN Taylor MK Hasegawa S Tilney NL Libby P. Interferon-Gamma Deficiency Prevents Coronary Arteriosclerosis But Not Myocardial Rejection in Transplanted Mouse Hearts. J Clin Invest (1997) 100(3):550–7. doi: 10.1172/jci119564 PMC508221 9239401

[16] Murayama H Takahashi M Takamoto M Shiba Y Ise H Koyama J. Deficiency of Tumour Necrosis Factor-Alpha and Interferon-Gamma in Bone Marrow Cells Synergistically Inhibits Neointimal Formation Following Vascular Injury. Cardiovasc Res (2008) 80(2):175–80. doi: 10.1093/cvr/cvn250 18791204

[17] Quaranta P Focosi D Freer G Pistello M. Tweaking Mesenchymal Stem/Progenitor Cell Immunomodulatory Properties With Viral Vectors Delivering Cytokines. Stem Cells Dev (2016) 25(18):1321–41. doi: 10.1089/scd.2016.0145 27476883

[18] Voisin C Cauchois G Reppel L Laroye C Louarn L Schenowitz C. Are the Immune Properties of Mesenchymal Stem Cells From Wharton’s Jelly Maintained During Chondrogenic Differentiation? J Clin Med (2020) 9(2):423. doi: 10.3390/jcm9020423 PMC7073626 32033151

[19] Chen C Hou J. Mesenchymal Stem Cell-Based Therapy in Kidney Transplantation. Stem Cell Res Ther (2016) 7:16. doi: 10.1186/s13287-016-0283-6 26852923 PMC4745166

[20] Leventhal JR Ildstad ST. Tolerance Induction in HLA Disparate Living Donor Kidney Transplantation by Facilitating Cell-Enriched Donor Stem Cell Infusion: The Importance of Durable Chimerism. Hum Immunol (2018) 79(5):272–6. doi: 10.1016/j.humimm.2018.01.007 29409743

[21] Li J Meng X Tao K Dou K. Prolongation of Cardiac Allograft Survival by Syngeneic Hematopoietic Stem/Progenitor Cell Transplantation in Mice. Adv Ther (2008) 25(9):935–42. doi: 10.1007/s12325-008-0091-1 18758696

[22] Casiraghi F Perico N Cortinovis M Remuzzi G. Mesenchymal Stromal Cells in Renal Transplantation: Opportunities and Challenges. Nat Rev Nephrol (2016) 12(4):241–53. doi: 10.1038/nrneph.2016.7 26853275

[23] Mannon RB. Macrophages: Contributors to Allograft Dysfunction, Repair, or Innocent Bystanders? Curr Opin Organ Transplant (2012) 17(1):20–5. doi: 10.1097/MOT.0b013e32834ee5b6 PMC3319132 22157320

[24] Di Ianni M Del Papa B De Ioanni M Moretti L Bonifacio E Cecchini D. Mesenchymal Cells Recruit and Regulate T Regulatory Cells. Exp Hematol (2008) 36(3):309–18. doi: 10.1016/j.exphem.2007.11.007 18279718

[25] Casiraghi F Azzollini N Cassis P Imberti B Morigi M Cugini D. Pretransplant Infusion of Mesenchymal Stem Cells Prolongs the Survival of a Semiallogeneic Heart Transplant Through the Generation of Regulatory T Cells. J Immunol (Baltimore Md 1950) (2008) 181(6):3933–46. doi: 10.4049/jimmunol.181.6.3933 18768848

[26] Ye K Lan X Wang G Zhang B Xu X Li X. B7-H1 Expression is Required for Human Endometrial Regenerative Cells in the Prevention of Transplant Vasculopathy in Mice. Stem Cells Int (2018) 2018:2405698. doi: 10.1155/2018/2405698 29731774 PMC5872625

[27] Huang WC Liao SK Wallace CG Chang NJ Lin JY Wei FC. Greater Efficacy of Tolerance Induction With Cyclosporine Versus Tacrolimus in Composite Tissue Allotransplants With Less Myeloablative Conditioning. Plast Reconstruct Surg (2011) 127(3):1141–8. doi: 10.1097/PRS.0b013e3182043695 21364417

[28] Buron F Perrin H Malcus C Héquet O Thaunat O Kholopp-Sarda MN. Human Mesenchymal Stem Cells and Immunosuppressive Drug Interactions in Allogeneic Responses: An In Vitro Study Using Human Cells. Transplant Proc (2009) 41(8):3347–52. doi: 10.1016/j.transproceed.2009.08.030 19857747

[29] Hoogduijn MJ Crop MJ Korevaar SS Peeters AM Eijken M Maat LP. Susceptibility of Human Mesenchymal Stem Cells to Tacrolimus, Mycophenolic Acid, and Rapamycin. Transplantation (2008) 86(9):1283–91. doi: 10.1097/TP.0b013e31818aa536 19005411

[30] Tsuji W Schnider JT McLaughlin MM Schweizer R Zhang W Solari MG. Effects of Immunosuppressive Drugs on Viability and Susceptibility of Adipose- and Bone Marrow-Derived Mesenchymal Stem Cells. Front Immunol (2015) 6:131. doi: 10.3389/fimmu.2015.00131 25932028 PMC4399413

[31] Hu C Li L. Melatonin Plays Critical Role in Mesenchymal Stem Cell-Based Regenerative Medicine In Vitro and In Vivo. Stem Cell Res Ther (2019) 10(1):13. doi: 10.1186/s13287-018-1114-8 30635065 PMC6329089

[32] Reiter RJ Tan DX Fuentes-Broto L. Melatonin: A Multitasking Molecule. Prog Brain Res (2010) 181:127–51. doi: 10.1016/s0079-6123(08)81008-4 20478436

[33] Jung FJ Yang L Harter L Inci I Schneiter D Lardinois D. Melatonin In Vivo Prolongs Cardiac Allograft Survival in Rats. J Pineal Res (2004) 37(1):36–41. doi: 10.1111/j.1600-079X.2004.00133.x 15230866

[34] Lin GJ Huang SH Chen YW Hueng DY Chien MW Chia WT. Melatonin Prolongs Islet Graft Survival in Diabetic NOD Mice. J Pineal Res (2009) 47(3):284–92. doi: 10.1111/j.1600-079X.2009.00712.x 19708865

[35] Vairetti M Ferrigno A Bertone R Rizzo V Richelmi P Bertè F. Exogenous Melatonin Enhances Bile Flow and ATP Levels After Cold Storage and Reperfusion in Rat Liver: Implications for Liver Transplantation. J Pineal Res (2005) 38(4):223–30. doi: 10.1111/j.1600-079X.2004.00193.x 15813898

[36] Inci I Inci D Dutly A Boehler A Weder W. Melatonin Attenuates Posttransplant Lung Ischemia-Reperfusion Injury. Ann Thorac Surg (2002) 73(1):220–5. doi: 10.1016/s0003-4975(01)03101-0 11834013

[37] Li Z Nickkholgh A Yi X Bruns H Gross ML Hoffmann K. Melatonin Protects Kidney Grafts From Ischemia/Reperfusion Injury Through Inhibition of NF-kB and Apoptosis After Experimental Kidney Transplantation. J Pineal Res (2009) 46(4):365–72. doi: 10.1111/j.1600-079X.2009.00672.x 19552759

[38] Calvo JR González-Yanes C Maldonado MD. The Role of Melatonin in the Cells of the Innate Immunity: A Review. J Pineal Res (2013) 55(2):103–20. doi: 10.1111/jpi.12075 23889107

[39] Zahran R Ghozy A Elkholy SS El-Taweel F El-Magd MA. Combination Therapy With Melatonin, Stem Cells and Extracellular Vesicles is Effective in Limiting Renal Ischemia–Reperfusion Injury in a Rat Model. Int J Urol (2020) 27(11):1039–49. doi: 10.1111/iju.14345 32794300

[40] Yu S Cheng Y Zhang L Yin Y Xue J Li B. Treatment With Adipose Tissue-Derived Mesenchymal Stem Cells Exerts Anti-Diabetic Effects, Improves Long-Term Complications, and Attenuates Inflammation in Type 2 Diabetic Rats. Stem Cell Res Ther (2019) 10(1):333. doi: 10.1186/s13287-019-1474-8 31747961 PMC6868748

[41] Mazini L Rochette L Amine M Malka G. Regenerative Capacity of Adipose Derived Stem Cells (Adscs), Comparison With Mesenchymal Stem Cells (Mscs). Int J Mol Sci (2019) 20(10):2523. doi: 10.3390/ijms20102523 PMC6566837 31121953

[42] Labat A Calise D Thiers JC Pieraggi MT Cerene A Fournial G. Simultaneous Orthotopic Transplantation of Carotid and Aorta in the Rat by the Sleeve Technique. Lab Anim (2002) 36(4):426–31. doi: 10.1258/002367702320389099 12396286

[43] Sho M Harada H Rothstein DM Sayegh MH. CD45RB-Targeting Strategies for Promoting Long-Term Allograft Survival and Preventingchronic Allograft Vasculopathy. Transplantation (2003) 75(8):1142–6. doi: 10.1097/01.Tp.0000060567.48258.9d 12717193

[44] Lee JH Han YS Lee SH. Potentiation of Biological Effects of Mesenchymal Stem Cells in Ischemic Conditions by Melatonin Via Upregulation of Cellular Prion Protein Expression. J Pineal Res (2017) 62(2):10.1111/jpi.12385. doi: 10.1111/jpi.12385 28095625

[45] Shaji AV Kulkarni SK Agrewala JN. Regulation of Secretion of IL-4 and IgG1 Isotype by Melatonin-Stimulated Ovalbumin-Specific T Cells. Clin Exp Immunol (1998) 111(1):181–5. doi: 10.1046/j.1365-2249.1998.00493.x PMC1904848 9472679

[46] Pasztorek M Rossmanith E Mayr C Hauser F Jacak J Ebner A. Influence of Platelet Lysate on 2D and 3D Amniotic Mesenchymal Stem Cell Cultures. Front Bioeng Biotechnol (2019) 7:338. doi: 10.3389/fbioe.2019.00338 31803733 PMC6873824

[47] Lan X Wang G Xu X Lu S Li X Zhang B. Stromal Cell-Derived Factor-1 Mediates Cardiac Allograft Tolerance Induced by Human Endometrial Regenerative Cell-Based Therapy. Stem Cells Transl Med (2017) 6(11):1997–2008. doi: 10.1002/sctm.17-0091 28941322 PMC6430050

[48] Wang H Qi F Dai X Tian W Liu T Han H. Requirement of B7-H1 in Mesenchymal Stem Cells for Immune Tolerance to Cardiac Allografts in Combination Therapy With Rapamycin. Transplant Immunol (2014) 31(2):65–74. doi: 10.1016/j.trim.2014.06.005 24978830

[49] Bartholomew A Sturgeon C Siatskas M Ferrer K McIntosh K Patil S. Mesenchymal Stem Cells Suppress Lymphocyte Proliferation In Vitro and Prolong Skin Graft Survival In Vivo. Exp Hematol (2002) 30(1):42–8. doi: 10.1016/s0301-472x(01)00769-x 11823036

[50] Illigens BM Yamada A Anosova N Dong VM Sayegh MH Benichou G. Dual Effects of the Alloresponse by Th1 and Th2 Cells on Acute and Chronic Rejection of Allotransplants. Eur J Immunol (2009) 39(11):3000–9. doi: 10.1002/eji.200838980 PMC2911804 19658090

[51] Syrjala SO Keranen MA Tuuminen R Nykanen AI Tammi M Krebs R. Increased Th17 Rather Than Th1 Alloimmune Response is Associated With Cardiac Allograft Vasculopathy After Hypothermic Preservation in the Rat. J Heart Lung Transplant (2010) 29(9):1047–57. doi: 10.1016/j.healun.2010.04.012 20591689

[52] Ye K Lan X Wang G Zhang B Xu X Li X. B7-H1 Expression is Required for Human Endometrial Regenerative Cells in the Prevention of Transplant Vasculopathy in Mice. Stem Cells Int (2018) 2018:1–12. doi: 10.1155/2018/2405698 PMC5872625 29731774

[53] Abele-Ohl S Leis M Mahmoudian S Weyand M Stamminger T Ensminger SM. Rag2-/- Gamma-Chain-/- Mice as Hosts for Human Vessel Transplantation and Allogeneic Human Leukocyte Reconstitution. Transpl Immunol (2010) 23(1-2):59–64. doi: 10.1016/j.trim.2010.04.003 20394817

[54] Rafat A Mohammadi Roushandeh A Alizadeh A Hashemi-Firouzi N Golipoor Z. Comparison of The Melatonin Preconditioning Efficacy Between Bone Marrow and Adipose-Derived Mesenchymal Stem Cells. Cell J (2019) 20(4):450–8. doi: 10.22074/cellj.2019.5507 PMC6099139 30123990

[55] Shaw KM Stern GM Sandler M. Melatonin and Parkinsonism. Lancet (London England) (1973) 1(7797):271. doi: 10.1016/s0140-6736(73)90118-9 4119422

[56] Gitto E Reiter RJ Sabatino G Buonocore G Romeo C Gitto P. Correlation Among Cytokines, Bronchopulmonary Dysplasia and Modality of Ventilation in Preterm Newborns: Improvement With Melatonin Treatment. J Pineal Res (2005) 39(3):287–93. doi: 10.1111/j.1600-079X.2005.00251.x 16150110

[57] Andersen LP Gogenur I Rosenberg J Reiter RJ. The Safety of Melatonin in Humans. Clin Drug Investig (2016) 36(3):169–75. doi: 10.1007/s40261-015-0368-5 26692007

[58] Fischbein MP Yun J Laks H Irie Y Fishbein MC Bonavida B. Role of CD8+ Lymphocytes in Chronic Rejection of Transplanted Hearts. J Thoracic Cardiovasc Surg (2002) 123(4):803–9. doi: 10.1067/mtc.2002.120008 11986610

[59] Luo J Zhang Z Sun H Song J Chen X Huang J. Effect of Melatonin on T/B Cell Activation and Immune Regulation in Pinealectomy Mice. Life Sci (2020) 242:117191. doi: 10.1016/j.lfs.2019.117191 31863775

[60] Di Nicola M Carlo-Stella C Magni M Milanesi M Longoni PD Matteucci P. Human Bone Marrow Stromal Cells Suppress T-lymphocyte Proliferation Induced by Cellular or Nonspecific Mitogenic Stimuli. Blood (2002) 99(10):3838–43. doi: 10.1182/blood.v99.10.3838 11986244

[61] Hamano K Bashuda H Ito H Shirasawa B Okumura K Esato K. Graft Vasculopathy and Tolerance: Does the Balance of Th Cells Contribute to Graft Vasculopathy? J Surg Res (2000) 93(1):28–34. doi: 10.1006/jsre.2000.5967 10945940

[62] Yuan X Paez-Cortez J Schmitt-Knosalla I D’Addio F Mfarrej B Donnarumma M. A Novel Role of CD4 Th17 Cells in Mediating Cardiac Allograft Rejection and Vasculopathy. J Exp Med (2008) 205(13):3133–44. doi: 10.1084/jem.20081937 PMC2605226 19047438

[63] Özdemir AT Özgül Özdemir RB Kırmaz C Sarıboyacı AE Ünal Halbutoğlları ZS Özel C. The Paracrine Immunomodulatory Interactions Between the Human Dental Pulp Derived Mesenchymal Stem Cells and CD4 T Cell Subsets. Cell Immunol (2016) 310:108–15. doi: 10.1016/j.cellimm.2016.08.008 27576175

[64] Park H Li Z Yang XO Chang SH Nurieva R Wang YH. A Distinct Lineage of CD4 T Cells Regulates Tissue Inflammation by Producing Interleukin 17. Nat Immunol (2005) 6(11):1133–41. doi: 10.1038/ni1261 PMC1618871 16200068

[65] Alvarez-Sanchez N Cruz-Chamorro I Lopez-Gonzalez A Utrilla JC Fernandez-Santos JM Martinez-Lopez A. Melatonin Controls Experimental Autoimmune Encephalomyelitis by Altering the T Effector/Regulatory Balance. Brain Behav Immun (2015) 50:101–14. doi: 10.1016/j.bbi.2015.06.021 26130320

[66] Yang XO Pappu BP Nurieva R Akimzhanov A Kang HS Chung Y. T Helper 17 Lineage Differentiation is Programmed by Orphan Nuclear Receptors ROR Alpha and ROR Gamma. Immunity (2008) 28(1):29–39. doi: 10.1016/j.immuni.2007.11.016 18164222 PMC2587175

[67] Ren W Liu G Chen S Yin J Wang J Tan B. Melatonin Signaling in T Cells: Functions and Applications. J Pineal Res (2017) 62(3):10.1111/jpi.12394. doi: 10.1111/jpi.12394 28152213

[68] Zhao Y Li X Yu D Hu Y Jin W Qin Y. Galectin-9 is Required for Endometrial Regenerative Cells to Induce Long-Term Cardiac Allograft Survival in Mice. Stem Cell Res Ther (2020) 11(1):471. doi: 10.1186/s13287-020-01985-0 33153471 PMC7643467

[69] Chen QH Wu F Liu L Chen HB Zheng RQ Wang HL. Mesenchymal Stem Cells Regulate the Th17/Treg Cell Balance Partly Through Hepatocyte Growth Factor In Vitro. Stem Cell Res Ther (2020) 11(1):91. doi: 10.1186/s13287-020-01612-y 32111238 PMC7049226

[70] Luz-Crawford P Hernandez J Djouad F Luque-Campos N Caicedo A Carrère-Kremer S. Mesenchymal Stem Cell Repression of Th17 Cells is Triggered by Mitochondrial Transfer. Stem Cell Res Ther (2019) 10(1):232. doi: 10.1186/s13287-019-1307-9 31370879 PMC6676586

[71] Mishima T Toda S Ando Y Matsunaga T Inobe M. Rapid Proliferation of Activated Lymph Node CD4(+) T Cells is Achieved by Greatly Curtailing the Duration of Gap Phases in Cell Cycle Progression. Cell Mol Biol Lett (2014) 19(4):638–48. doi: 10.2478/s11658-014-0219-z PMC6275717 25424911

[72] Blattman JN Antia R Sourdive DJ Wang X Kaech SM Murali-Krishna K. Estimating the Precursor Frequency of Naive Antigen-Specific CD8 T Cells. J Exp Med (2002) 195(5):657–64. doi: 10.1084/jem.20001021 PMC2193761 11877489

[73] Ribeiro A Laranjeira P Mendes S Velada I Leite C Andrade P. Mesenchymal Stem Cells From Umbilical Cord Matrix, Adipose Tissue and Bone Marrow Exhibit Different Capability to Suppress Peripheral Blood B, Natural Killer and T Cells. Stem Cell Res Ther (2013) 4(5):125. doi: 10.1186/scrt336 24406104 PMC3854702

[74] Luque-Campos N Contreras-López RA Jose Paredes-Martínez M Torres MJ Bahraoui S Wei M. Mesenchymal Stem Cells Improve Rheumatoid Arthritis Progression by Controlling Memory T Cell Response. Front Immunol (2019) 10:798. doi: 10.3389/fimmu.2019.00798 31040848 PMC6477064

[75] Baaten BJ Tinoco R Chen AT Bradley LM. Regulation of Antigen-Experienced T Cells: Lessons From the Quintessential Memory Marker Cd44. Front Immunol (2012) 3:23. doi: 10.3389/fimmu.2012.00023 22566907 PMC3342067

[76] Roldan C Mirabet S Brossa V Molto E Lopez L Alvaro Y. Correlation of Immunological Markers With Graft Vasculopathy Development in Heart Transplantation. Transplant Proc (2012) 44(9):2653–6. doi: 10.1016/j.transproceed.2012.09.048 23146484

[77] Medrano-Campillo P Sarmiento-Soto H Alvarez-Sanchez N Alvarez-Rios AI Guerrero JM Rodriguez-Prieto I. Evaluation of the Immunomodulatory Effect of Melatonin on the T-cell Response in Peripheral Blood From Systemic Lupus Erythematosus Patients. J Pineal Res (2015) 58(2):219–26. doi: 10.1111/jpi.12208 25612066

[78] Zhao CN Wang P Mao YM Dan YL Wu Q Li XM. Potential Role of Melatonin in Autoimmune Diseases. Cytokine Growth Factor Rev (2019) 48:1–10. doi: 10.1016/j.cytogfr.2019.07.002 31345729

[79] Koga T Ichinose K Mizui M Crispín JC Tsokos GC. Calcium/Calmodulin-Dependent Protein Kinase IV Suppresses IL-2 Production and Regulatory T Cell Activity in Lupus. J Immunol (Baltimore Md 1950) (2012) 189(7):3490–6. doi: 10.4049/jimmunol.1201785 PMC3448834 22942433

[80] Sakaguchi S Ono M Setoguchi R Yagi H Hori S Fehervari Z. Foxp3+ CD25+ CD4+ Natural Regulatory T Cells in Dominant Self-Tolerance and Autoimmune Disease. Immunol Rev (2006) 212:8–27. doi: 10.1111/j.0105-2896.2006.00427.x 16903903

[81] Zorn E. Effector B Cells in Cardiac Allograft Vasculopathy. Curr Opin Organ Transplant (2019) 24(1):31–6. doi: 10.1097/mot.0000000000000591 PMC8682961 30480642

[82] Jansen MA Otten HG de Weger RA Huibers MM. Immunological and Fibrotic Mechanisms in Cardiac Allograft Vasculopathy. Transplantation (2015) 99(12):2467–75. doi: 10.1097/tp.0000000000000848 26285017

[83] Corcione A Benvenuto F Ferretti E Giunti D Cappiello V Cazzanti F. Human Mesenchymal Stem Cells Modulate B-cell Functions. Blood (2006) 107(1):367–72. doi: 10.1182/blood-2005-07-2657 16141348

