# Corrigendum: Melatonin Synergizes With Mesenchymal Stromal Cells Attenuates Chronic Allograft Vasculopathy

**DOI:** 10.3389/fimmu.2021.741234

**Published:** 2021-09-10

**Authors:** Ya-fei Qin, De-jun Kong, Hong Qin, Yang-lin Zhu, Guang-ming Li, Cheng-lu Sun, Yi-ming Zhao, Hong-da Wang, Jing-peng Hao, Hao Wang

**Affiliations:** ^1^Department of General Surgery, Tianjin Medical University General Hospital, Tianjin, China; ^2^Tianjin General Surgery Institute, Tianjin Medical University General Hospital, Tianjin, China; ^3^Department of Anorectal Surgery, The Second Hospital of Tianjin Medical University, Tianjin, China

**Keywords:** chronic allograft vasculopathy, melatonin, mesenchymal stromal cells, aorta transplantation, mice

In the original article, there was a mistake in **Figure 3A** as published. The figure was placed repeatedly in the process of copying/pasting our original images. The corrected **Figure 3A** appears below. In the original article, there was a mistake in **Figure 6A** as published. The figure was placed repeatedly in the process of copying/pasting our original images. The corrected **Figure 6A** appears below.

**Figure 3:**
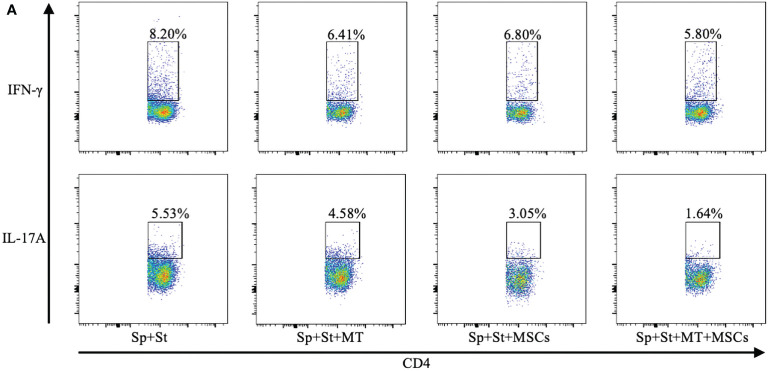
**(A)** The pseudocolor of Th1 (CD4+IFN-γ+) cells and Th17 (CD4+IL-17A+) cells *in vitro*.

**Figure 6:**
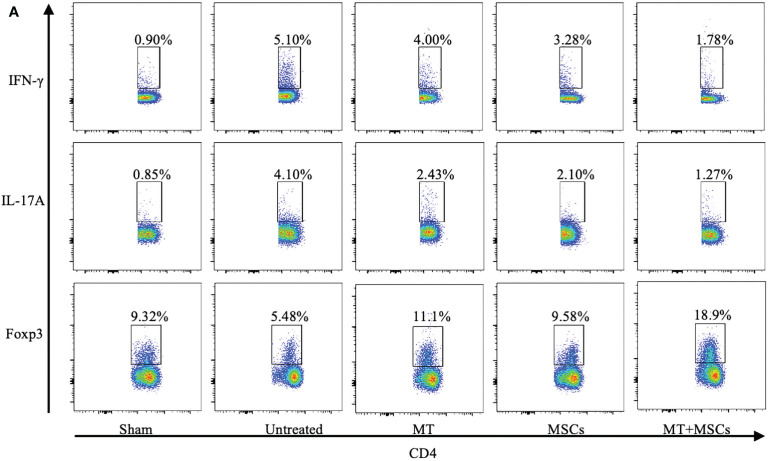
**(A)** The pseudocolor of Th1 (CD4+IFN-γ+) cells, Th17 (CD4+IL-17A+) cells, and CD4+Foxp3+ Tregs *in vivo*.

The authors apologize for these errors and state that this does not change the scientific conclusions of the article in any way. The original article has been updated.

## Publisher’s Note

All claims expressed in this article are solely those of the authors and do not necessarily represent those of their affiliated organizations, or those of the publisher, the editors and the reviewers. Any product that may be evaluated in this article, or claim that may be made by its manufacturer, is not guaranteed or endorsed by the publisher

